# When learning becomes doing: contributions of the next generation of scientists to knowledge about genetics and genomics

**DOI:** 10.1093/g3journal/jkaf273

**Published:** 2026-01-07

**Authors:** Lauren M McIntyre

**Affiliations:** Department of Molecular Genetics and Microbiology, University of Florida Genetics Institute, University of Florida, PO Box 100266, Gainesville, FL 32610, United States

Genetics and genomics education has undergone a transition from demonstrating science to participating in science. At G3 we welcome these exciting contributions from teams of established and emerging scientists. The first of these papers arose from a large-scale project, the Genomics Education Partnership (GEP) program, which nearly a decade ago, sequenced and annotated genes on the *D. erecta*, *D. mojavensis*, and *D. grimshawi* Muller F elements and euchromatic domains from the Muller D element (Leung et al. 2015). This paper, with 1014 authors, including more than 900 undergraduate students from 63 higher education institutions, set the stage for a revolution. Led by Sally Elgin, this innovative team advanced science, learned how to handle the messiness of real data, how to evaluate different kinds of evidence, and how to justify their conclusions. The team continued its successful model with characterization of Muller's F on the *D. ananassae* genome (Leung et al. 2017).

As sequencing technology continues to advance, it has been coupled with the development of reproducible informatics pipelines. These innovations have fueled an increase in the scale of the science that can be accomplished with a multi-layered model for engagement of graduate student teaching assistants, postdoctoral associates, and faculty with undergraduate learners. The American Campus Tree Genomes (ACTG) initiative is an NSF-funded project led by Alex Harkess whose whole genome sequences includes the Washington State University apple (Zhang et al. 2024) and the Auburn University pear (Yocca et al. 2024), and expanded to animals under the leadership of Ellie Armstrong with a sister project, Campus Mascot Genomes, which has sequenced the genome of the Middlebury College Grey Fox (Armstrong et al. 2024). A separate effort in an integrated undergraduate and graduate student training program in biodiversity and conservation genomics, led to the assembly and annotation of the threatened North American walnut (Guzman-Torres et al. 2024).

Another successful avenue for engagement has been in teaching students how to properly conduct a genetic screen, as demonstrated in Drosophila: using an expression-based cell lineage analysis and for a class phenotype in “A genetic screen of transcription factors in the Drosophila melanogaster abdomen identifies novel pigmentation genes” (Olson et al. 2019; Petrosky et al. 2024). Screens in Mycobacterium, supported by the HHMI-sponsored SEA-GENES project have identified growth inhibitors encoded by mycobacteriophage (Heller and Sivanathan 2022; Amaya et al. 2023; Pollenz et al. 2024; Tafoya et al. 2025).

G3 also supports the publication of molecular tools developed to make deploying high-throughput screening in an education setting more robust and efficient, enabling this outreach to extend beyond the University setting into secondary school classrooms (Chang et al. 2022; Rankin et al. 2024; Yeager et al. 2025). At the same time, we recognize that engagement doesn’t need to be limited to the classroom. Scientists at the University of Washington characterized the genomic makeup of a *Saccharomyces cerevisiae* ale yeast widely used in the production of Hefeweizen beers, and in the process created a resource to guide fermentation protocols, strain handling, and engineering practices in commercial brewing and fermentation environments (Garge et al. 2024). Read more about this project in the Genes to Genomes blog.

G3 welcomes contributions from collectives, and supports community efforts to develop novel reagents and bioinformatic tools that enable scientists to engage more broadly with our community so that, ultimately, more people can participate in and contribute to the creation of foundational knowledge in genetics and genomics. The value of this work is apparent in the online accesses and citations these papers receive!

As an editor, seeing the excellent papers whose novel findings were made possible by these communal efforts buoys my spirits and gives me hope for the future. If you have engaged in one of these projects and would like to publish your work, or you are developing robust and reproducible workflows that support these efforts, our Reports format is designed to make communicating your success straightforward. I hope to see more of these submissions in 2026!
